# Protein Expression During Murine Thymus Differentiation

**DOI:** 10.1155/1996/64697

**Published:** 1996

**Authors:** Johann Rudolf Frey, Klaus-Ulrich Hartmann, Ivan Lefkovits

**Affiliations:** 1 Basel Institute for Immunology P.O. Box Grenzacherstrasse 487 Basel CH-4005 Switzerland; 2 Institute for Experimental Immunology Philipps University Marburg Germany

**Keywords:** Database, image analysis, protein expression, thymus development, two-dimensional gel electrophoresis

## Abstract

Driven by our long-standing interest in identifying proteins of the immune system and in characterizing processes involved in lymphocyte differentiation, we studied protein expression in biosynthetically labeled fetal and newborn thymus by 2D gel electrophoresis. Autoradiographs of the gels were scanned with a densitometer and image analysis was performed using the *Kepler* system. Calibrated polypeptide spot abundances (volumes) were compared to assesses qualitative and quantitative changes of the spot volumes. Among over 300 proteins evaluated at GD (gestation day) 13,15, and 17, there were sets of proteins that increased and others that decreased in intensity. We could in addition recognize proteins that were completely absent at GD 13 and/or 15 and that appeared thereafter to gradually increase in intensity. Conversely, various polypeptide spots present at early stages (at GD 13 and 15) disappear later (at GD 17 or at birth). Among the proteins that increase in intensity prevail molecules with masses less than 35 kD, whereas a considerable portion of those that decrease in intensity are characterized by masses above 60 kD. Spots reported in this communication were not defined beyond tagging them with numbers, which is a prerequisite to follow them up in the proteinpaedia developed in our laboratory. The next step will be to retrieve the coding sequences from the existing partitioned cDNA library (BW 5147) as well as from thymocyte subtraction libraries. We predict that among those polypeptides with varying intensity, important regulatory proteins in thymus development will be found.


*Developmental Immunology*, 1996, Vol 5, pp. 53–66
Reprints available directly from the publisher
Photocopying permitted by license only

© 1996 OPA (Overseas Publishers Association)
Amsterdam B.V. Published in The Netherlands
by Harwood Academic Publishers GmbH
Printed in Singapore

### Protein Expression During Murine Thymus Differentiation

JOHANN RUDOLF FREY,*† KLAUS-ULRICH HARTMANN,‡ and IVAN LEFKOVITS†

†*Basel Institute for Immunology, P.O. Box, Grenzacherstrasse 487, CH-4005 Basel, Switzerland*
‡*Institute for Experimental Immunology, Philipps University, Marburg, Germany*

Driven by our long-standing interest in identifying proteins of the immune system and in characterizing processes involved in lymphocyte differentiation, we studied protein expression in biosynthetically labeled fetal and newborn thymus by 2D gel electrophoresis. Autoradiographs of the gels were scanned with a densitometer and image analysis was performed using the *Kepler* system. Calibrated polypeptide spot abundances (volumes) were compared to assesses qualitative and quantitative changes of the spot volumes. Among over 300 proteins evaluated at GD (gestation day) 13, 15, and 17, there were sets of proteins that increased and others that decreased in intensity. We could in addition recognize proteins that were completely absent at GD 13 and/or 15 and that appeared thereafter to gradually increase in intensity. Conversely, various polypeptide spots present at early stages (at GD 13 and 15) disappear later (at GD 17 or at birth). Among the proteins that increase in intensity prevail molecules with masses less than 35 kD, whereas a considerable portion of those that decrease in intensity are characterized by masses above 60 kD. Spots reported in this communication were not defined beyond tagging them with numbers, which is a prerequisite to follow them up in the proteinpaedia developed in our laboratory. The next step will be to retrieve the coding sequences from the existing partitioned cDNA library (BW 5147) as well as from thymocyte subtraction libraries. We predict that among those polypeptides with varying intensity, important regulatory proteins in thymus development will be found.

KEYWORDS: Database, image analysis, protein expression, thymus development, two-dimensional gel electrophoresis.

### INTRODUCTION

The thymus is the central organ for the differentiation and maturation of T lymphocytes. In murine embryogenesis, the thymus anlage originates from endodermal and ectodermal epithelial tissue, separating from the third pharyngeal arch around day 9 of gestation (GD 9). New vascularization and innervation takes place in the thymus anlage after its descent into the mediastinum. At approximately GD 11, dendritic cells and lymphoid precursor cells begin to immigrate and to colonize the thymus anlage. Thymic epithelial cells followed by dendritic cells are then among the first cells in the fetus to express MHC antigens. The immigrating lymphoid precursor cells as well as the sequence of their maturation in the thymus—including early initial phenotypic changes, rearrangement and expression of specific receptors, selection and differentiation—have been well described (Scollay, 1980; Boyd and Hugo, 1991; Van Ewijk et al., 1994; Kisielow and von Boehmer, 1995). By GD 18, the first mature T-cell receptor positive cells emigrate from the thymus (Scollay and Godfrey, 1995; Tough and Sprent, 1995) and populate the peripheral lymphoid organs. Although intensive cellular and paracrine interactions guide the differentiation and maturation of the thymocytes, only few of the participating intracellular regulatory elements are described (Ivanov et al., 1993; Sen et al., 1994; Xie and Palacios, 1994). Therefore, a more complete knowledge of the molecules involved will be necessary to understand both the normal regulation and defective mechanisms occurring in T-cell development.

In the work described in this paper, we analyzed preparations of total proteins from embryonic and neonatal thymus tissue by 2D gel electrophoresis. Typically, this technique allows the study of several hundred polypeptides in a single experimental run

*Corresponding author.

collectively (Anderson and Anderson, 1978). Apart from qualitative aspects of the information, the emphasis of our study was on the quantitative assessment of the kinetic behavior of thymic proteins in the mouse between GD 13 and birth. To achieve this, we used computer-aided image analysis of the autoradiographs as described elsewhere (Anderson and Anderson, 1978; Lefkovits et al., 1988; Lefkovits et al., 1995a).

### RESULTS

### The Development of the Thymus During Embryogenesis

The purpose of this study was to determine changes in the overall polypeptide composition of the fetal thymus at times known for important developmental processes. The following developmental stages can be described: At GD 11 to 12, the thymus begins

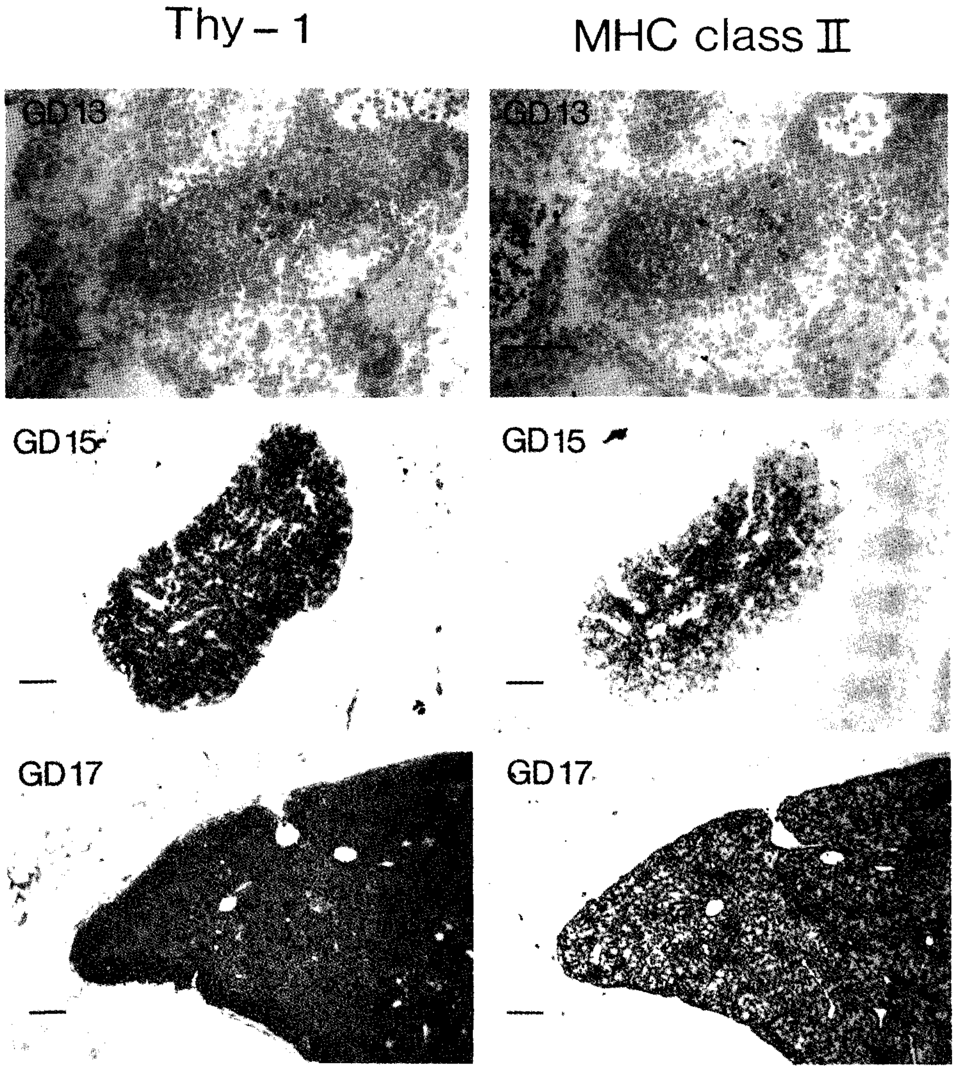

FIGURE 1. Cryosections of embryonic thymuses. The mass of the thymus increases from GD 13 through GD 15 and GD 17 approximately by 3-fold and 50-fold, respectively. Cryosections of whole mouse fetuses at GD 13, 15, and 17 (top, middle, and bottom micrographs) were stained with either anti-Thy-1 (30H12, left side) or anti-MHC class II (P7/7, right side). Detection was carried out using biotinylated goat-anti-rat Ig and streptavidin-peroxidase. The bars represent 0.2 mm.

to be histologically recognizable, and by GD 13, the thymic tissue appears more clearly demarcated from its surroundings. At GD 15, the earliest TCR rearrangements are detectable, and at GD 17, all stages of T-cell maturation can be followed phenotypically. Already at birth, substantial numbers of mature thymocytes leave the thymus and populate the periphery.

After thymus colonization with lymphoid (Jotereau et al., 1987; Imhof et al., 1991; Palacios and Samaridis, 1991) and dendritic (Ardavin et al., 1993) precursor cells, initial rounds of proliferation and differentiation occur. In order to design a meaningful experiment, we had to gain certainty that we can dissect not only the fetal thymus gland, but that we are working with tissue accessible to a satisfactory biosynthetic labeling. Although some data exist on the number of thymocytes present in the thymus during the progression of differentiation, we had to verify experimentally that we are dealing with a workable model. Cryosections of fetal thymuses of different stages of development had been stained with antibodies directed against Thy-1 and against MHC class II antigens. By GD 13, some Thy-1- and MHC class II–positive lymphoid cells are seen (Fig. 1, top two micrographs), dendritic cells being still rare at this time; MHC class II antigen can be detected in the developing thymus earlier than anywhere else in the fetus. At GD 15, a rather complete network of dendritic cells fills the thymus; thereafter, the number of cells positive for both markers increases rapidly, and at GD 17, already 98% of the lymphoid thymocytes are Thy-1-positive. Most of the thymocytes now coexpress TCR, CD4, and CD8 molecules as well. We attempted to calculate the number of thymocytes in the thymus gland at the gestation days under consideration. For GD 13—when integrating the counts of the cryosections—there are about 1000 thymocytes in the thymus gland, while the counts for GD 15 and 17 are $10^4$ and $10^7$, respectively. The bulk of the thymic volume for the three gestation times is approximately 0.02 $mm^3$, 0.06 $mm^3$, and 1 $mm^3$. Thus for all three gestation times, even when the number of cells is on the order of 1000 per thymus gland, there should result 2D gel patterns of interpretable quality. The sample size is large enough to ensure reproducible results. We have performed several parallel cultures obtaining essentially the same patterns.

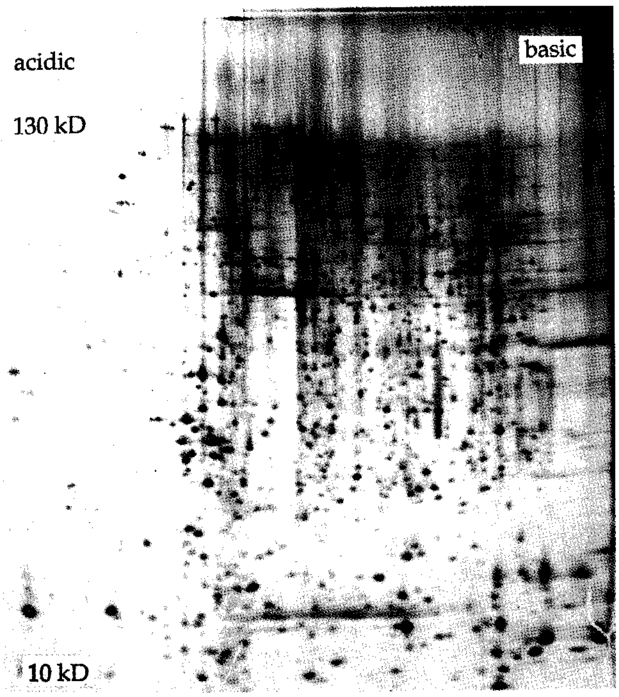

FIGURE 2. Autoradiograph of a 2D gel prepared from GD 17 thymus biosynthetically labeled *in vitro*. The solubilized sample was separated according to charge (horizontal axis) and size (vertical axis) in the ISODALT system (see Materials and Methods). The gel was dried and exposed for 12 days to an XAR-5 film.

### Polypeptide Pattern of the Fetal Thymus Lobes

In order to establish optimal labeling conditions, we compared autoradiographs of 2D gels prepared from *in vivo* and *in vitro* labeled GD 17 thymus, as described in Materials and Methods. From this set of experiments (data not shown), we have concluded that both approaches (*in vivo* and *in vitro* labeling) lead to similar protein patterns. Thus, for reasons of ease of manipulation and efficiency of labeling, we have opted for all further experiments to use *in vitro* labeling. In Fig. 2, we show the photograph of a 2D gel from a thymus at GD 17. On this gel, approximately 700 spots are discernible covering a broad range of molecular masses (10 to 130 kD) and a wide range of charges (pI 3 to 8). After spot modeling and matching, the image of the GD 17 autoradiograph contains 550 evaluable spots.

### 2D Gel Electrophoresis Patterns of Thymus Lobes from GD 13 to Birth

It is quite obvious that the previously mentioned differentiation events underlie changes at the molecular level. We argued that the results of these changes—visualized by 2D gels—will reveal proteins, the expression of which is turned on or off as thymus differentiation proceeds from one gestation stage to another.

The 2D gels, obtained from the labeled samples as explained in Materials and Methods, were exposed to X-ray film and the films were scanned and subjected to image analysis. The analyzed image is referred to as a spot "file", which is a list of data on the spots, while its graphical representation is called a spot "pattern," which is an idealized model of the original gel image. The spot pattern is first edited in order to remove artifacts (e.g., smears, streaks, or false spots), and then the images are "matched." The process of matching involves, first, the choice of a so-called master pattern, which in most instances is derived from one of the experimental patterns, and, second, the assignment of a numbering system to the master pattern such that each spot of the master is identified by a master spot number. Then by a combination of interactive and automatic matching, congruent spots in all analyzed patterns receive the same spot numbers.

In this project with an emphasis on quantitative information, the spots of each gel were calibrated, keeping the sum of total intensity of all spots constant. The calibration step led to changes of spot abundance (spot volume) amounting to less than ±3%. Such matched and calibrated spot files are fully comparable and spot intensities can be evaluated. We have attempted to visualize spots that are profoundly altered during the process of differentiation observed at GD 13, 15, and 17. This can be seen in Figs 3 and 4, in which several features of the experiment are apparent. In panel A, there are the full gel images represented (GD 17 in Fig. 3 and GD 13 in Fig. 4); the frames within the upper images are congruent with similar views taken from GD 13, 15, and 17 (panel B). The filled ellipses with numbers indicate nine spots that fulfill the criteria of either increase (Fig. 3B) or decrease (Fig. 4B) in intensities throughout the measurements. In panel C, for the indicated spots, there are histograms shown in which the relative spot intensity alterations are depicted. From the total of 312 spots, occurring at GD 13, 15, and 17, there were 83 spots increasing and 95 decreasing in intensity throughout the observation period.

The previously described analysis of the three experimental time points (GD 13 to 17) was extended to include the remaining gestation period up to birth, during which quite a profound emigration of mature thymocytes occurs (Scollay, 1980) Such a completed picture is represented in Figs 5 and 6 for increasing and decreasing spots, respectively. There are 29 spots (of 272) that increase up to birth and 30 spots (of 272) belonging to the category of decreasing spots. As expected, there are many spots that do not change in intensity (18 spots) during the longer observation period (from GD 13 to birth), as indicated in the histograms of Fig. 7.

The results described so far were compiled in such a way that only spots present at all three or four time points of the two observation periods were considered. This is obviously only the first approximation of the events, because we can assume that proteins that are not yet produced at GD 13, will appear later and then increase in abundance, or alternatively there exist proteins that are produced on day 13, decrease thereafter, and disappear completely. Such spots might in fact be of great importance, because they represent polypeptides that are required or permitted only at certain stages of differentiation. In Fig. 8A six spots are depicted that are absent at GD 13 and 15 and appear thereafter. The counterpart of this event is a set of thirteen spots that is present at GD 13 and 15 and is absent by GD 17 (Fig. 8B). In Fig. 8C, eight spots are represented that appear only at GD 15 and increase thereafter. The converse set of spots that is decreasing from GD 13 to 17 and lacking at birth was already considered as a subset of those data forming the image in Fig. 4A.

In Table 1, quantitative information on the spots from all four patterns is presented. The number of detected spots varies throughout the whole observation period (range 429 to 550), and because some spots appear and others disappear, the total number of spots in the master pattern is considerably higher (764) than in any of the experimental patterns.

The sets of spots common to all patterns within an observation period, on one hand, 312 spots within the three fetal patterns and, on the other hand, 272

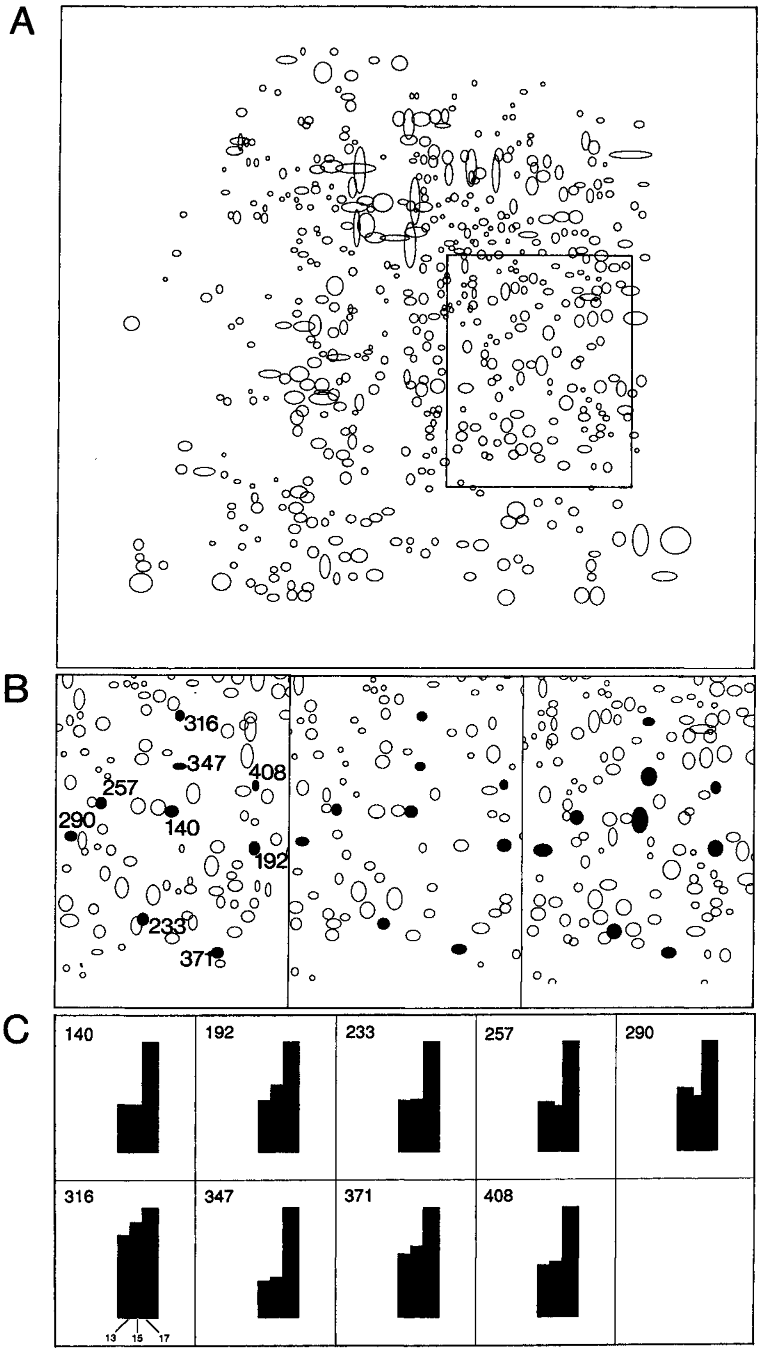

FIGURE 3. Polypeptides in thymuses that increase in abundance during gestation. In part A, the computer-modeled 2D gel pattern from a thymic sample at GD 17 is shown. The frame indicates a gel region that is displayed at higher magnification in part B. Here portions of patterns GD 13, 15, and 17 are displayed. Nine spots increasing in abundance are highlighted (filled ellipses) and the corresponding spot numbers indicated. In part C, histograms for these nine spots are shown. Each histogram consists of three bars (for patterns GD 13, 15, and 17). The height of the bar reflects the relative spot intensity (called spot volume). Spot 347, for example, increases only marginally between GD 13 and GD 15 but increases strongly in intensity between GD 15 and GD 17. Note that there are many more spots belonging to the "increasing" category, but they are outside of the magnified panels and hence not displayed.

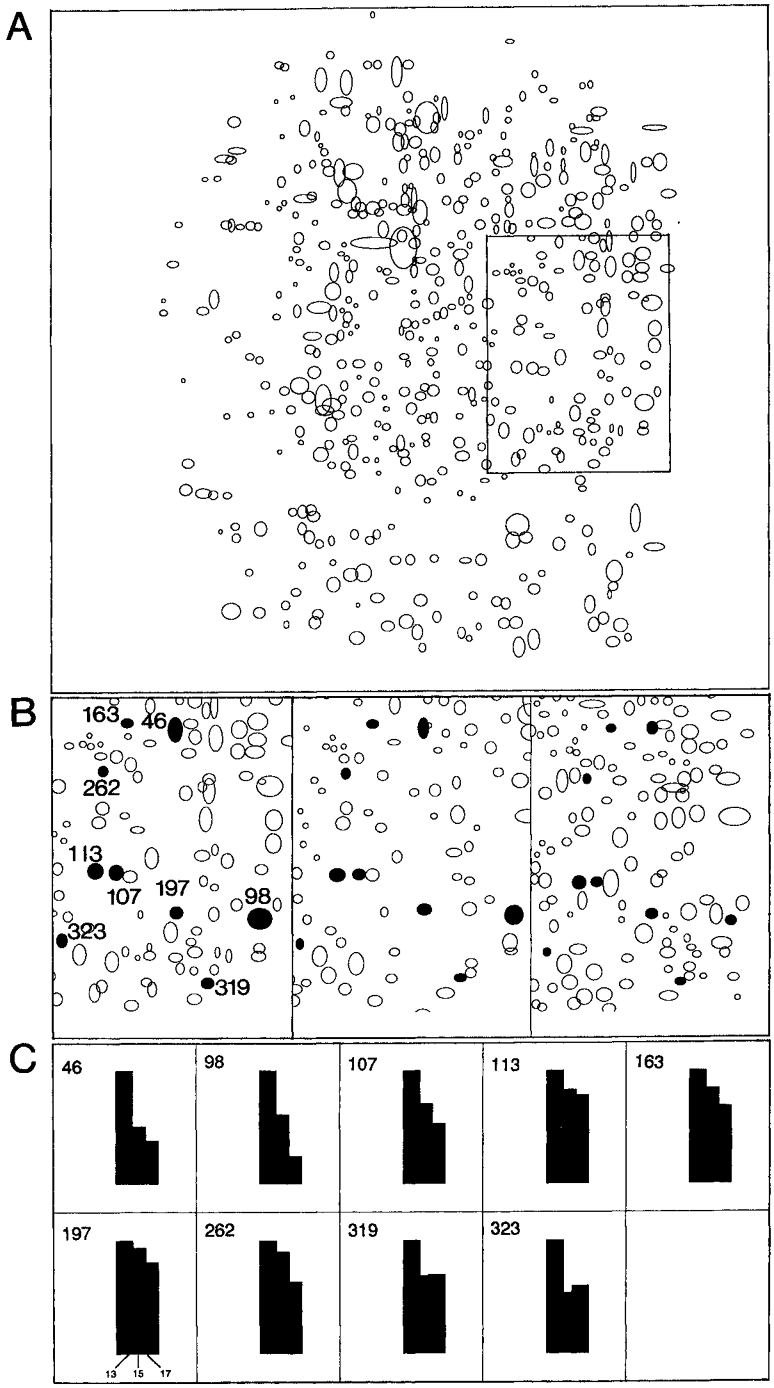

FIGURE 4. Polypeptides in thymuses that decrease in abundance during gestation. Figure presentation is identical to that one shown in Fig. 3, though the pattern in part A reflects GD 13 and the position of the frame is slightly shifted within the gel. Nine spots are marked that decrease in intensity. For example, spot 98 is very intense at GD 13 and decreases rapidly by GD 15.

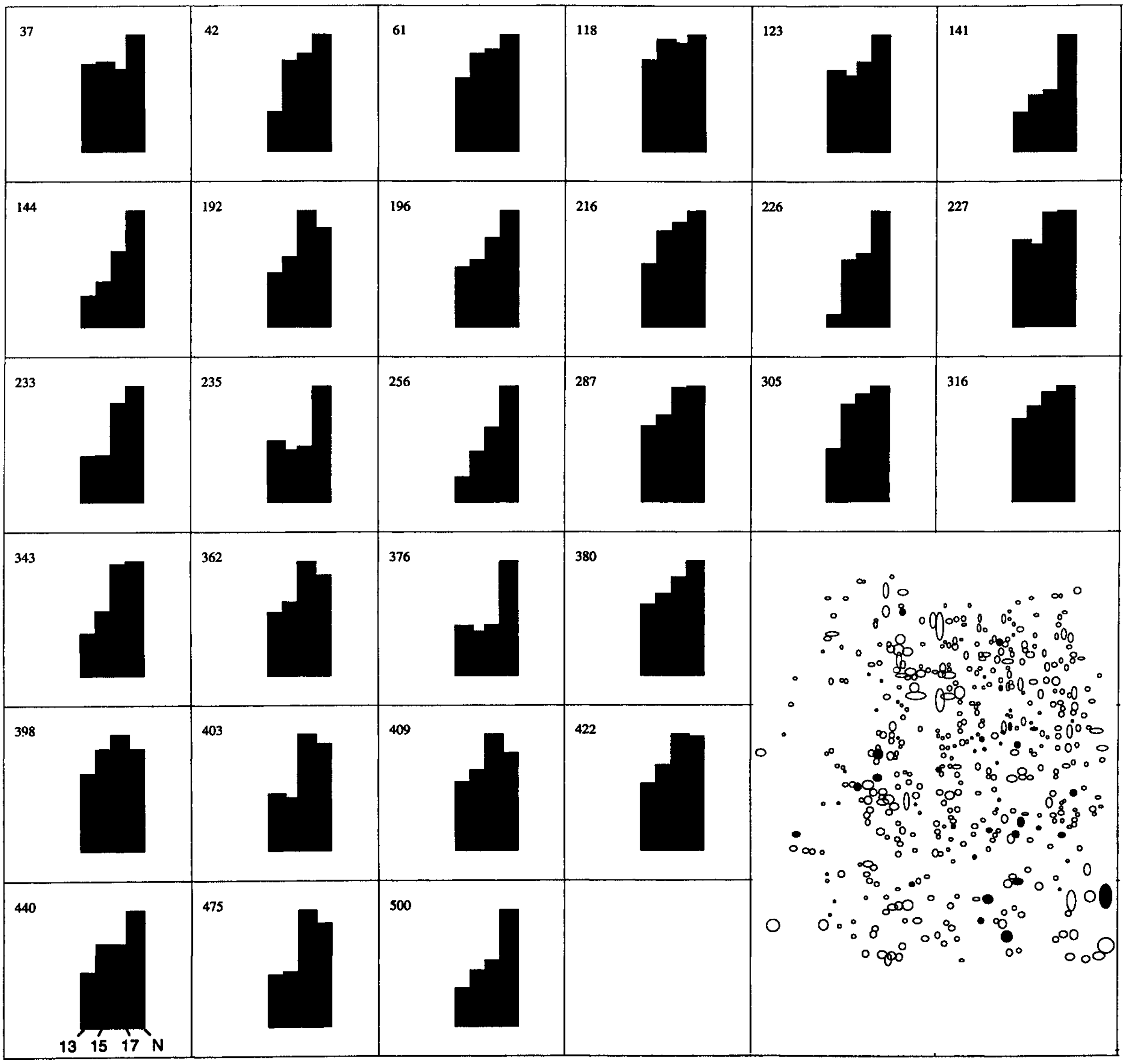

FIGURE 5. Polypeptides in thymuses that increase in abundance during gestation and up to birth. From the total number of 429 spots present in the overall pattern (newborn, shown at the lower right corner), there are 29 spots that increase from GD 13 up to birth. The histogram consists of four bars—GD 13, 15, 17, and newborn. The numbers are presented in a font size that is not well readable in this display.

spots within all four patterns, from GD 13 through newborn, were used to recognize those spots with changing or stable abundance. As this presentation emphasizes dynamic changes in spot intensities (volumes), it is of importance to realize that the mean intensity of the spots is quite stable throughout the whole observation period.

Furthermore, the most common 50% of spots have volumes of about 2000 to 9000 units. The spots belonging to the category of the 10% smallest spots have volumes below 1400 units and the 10% of the largest spots have volumes above 12,700 units (for GD 13 and higher values for later stages). The sum of all spot volumes is fluctuating within a narrow range (±10%) and the number itself was needed for the determination of calibration factors.

### DISCUSSION

The 2D gel analysis of the samples from thymuses at various gestation days yielded a large amount of

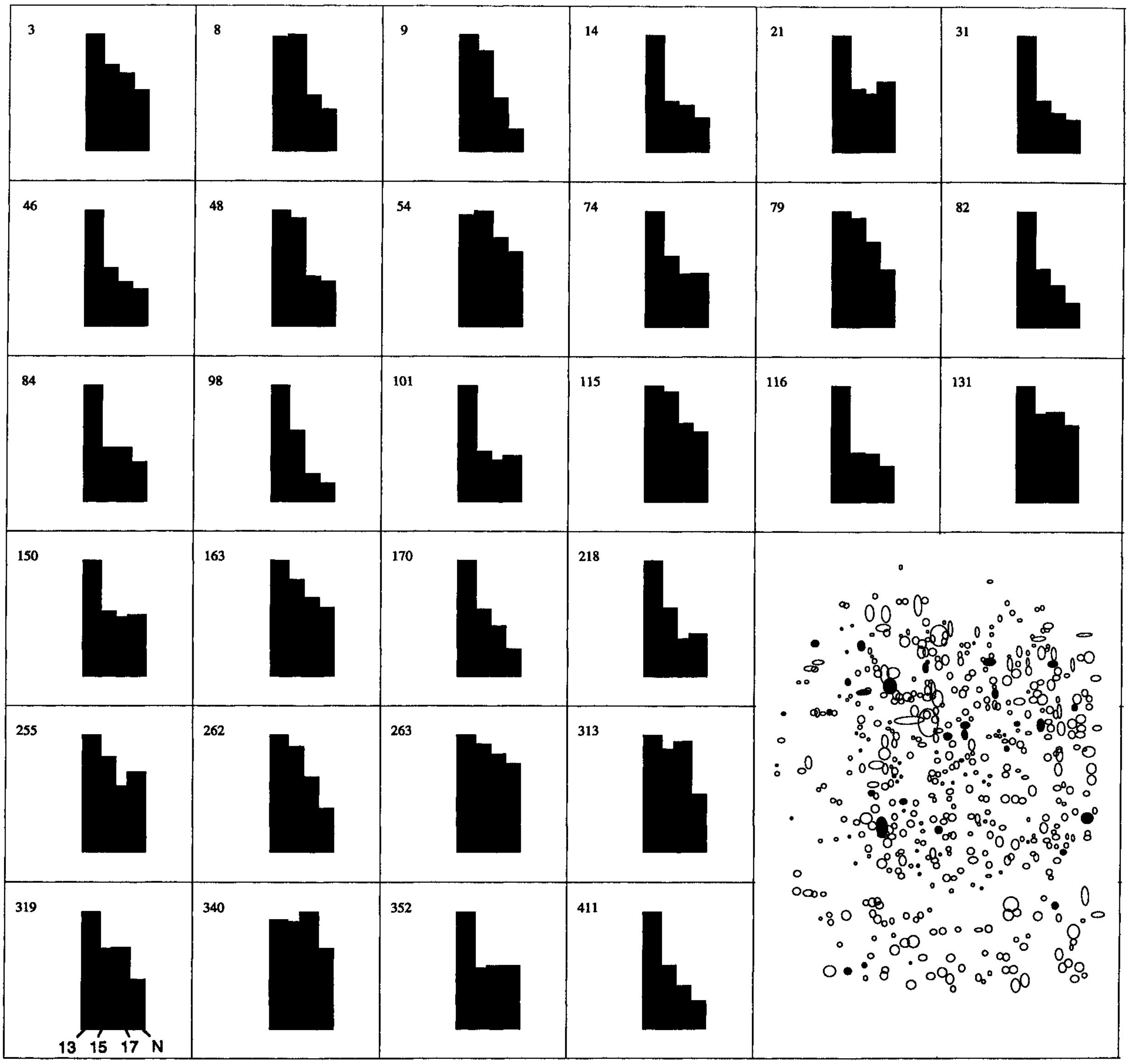

FIGURE 6. Polypeptides in thymuses that decrease in abundance during gestation and up to birth. As in Fig. 5, the whole pattern (GD 13; 505 spots in total) is shown at the lower right corner. The histograms represent 30 marked spots.

data, which can be used in at least two different manners: one can learn about the dynamic changes in the protein expression during differentiation, and one can pinpoint to polypeptide spots for which further scrutiny (including gene retrieval) would be appropriate. Before attempting to interpret the results, one should ask, how meaningful the changes in the observed patterns are. We analyzed changes in thymus lobes rather than in isolated thymocytes; thus, the changes that occur in the thymic stroma will be superimposed on the changes in the thymocytes. Nevertheless, given the relative abundance of thymocytes, we assume that most of the changes in the protein patterns are thymocyte-related. Changes in protein expression originating from the thymic stroma might be interesting, as profoundly as those in thymocytes, and it will be meaningful to distinguish them from each other.

Labeling of thymocytes is not sufficiently efficient in full medium and therefore $^{35}$S-methionine is added to cultures depleted of cold methionine. Under the latter condition, thymocytes might be driven to the

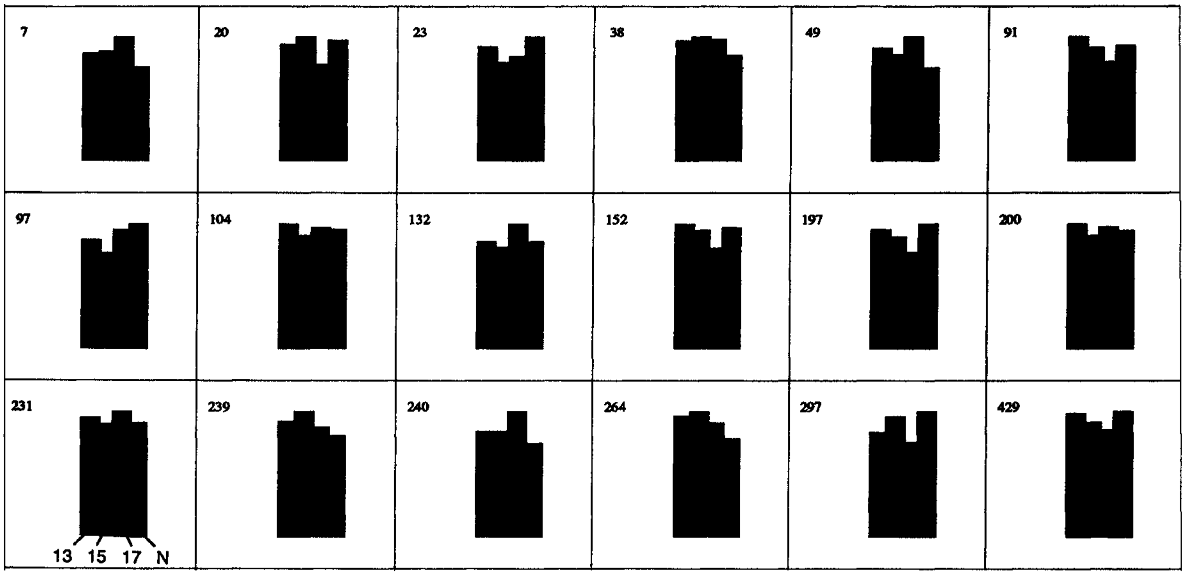

FIGURE 7. Polypeptides in thymuses that are constant in abundance during gestation and up to birth. The histograms stand for 18 spots that are constant in abundance throughout the gestation and up to birth. Each histogram, similarly to Figs 5 and 6, consist of four bars—GD 13, 15, 17, and newborn.

expression of some proteins that would not be synthesized under physiological conditions.

About 27% of all spots increase and 30% decrease in intensity, if one considers the three gestation days (GD 13, 15, and 17). These percentages refer to spots that are present at all three time points. Further, there are about 6% of spots that are constant throughout the observation period of the three gestation days. The remaining 37% of spots did not meet the criteria for any of the three aforementioned kinetics due to aberrant spot intensity at GD 15. Although we are interested in defining the changes throughout the fetal development up to birth, we were cautious to keep the comparison with the data on the newborns separate, because quantitative comparisons are less certain to be correct at the time when efflux of thymocytes is accelerated.

As can be seen in Table 1, the number of spots common to all four patterns (272) is smaller than the corresponding one for the three fetal time points (312). Accordingly, the sets of spots that increase and decrease (11% for both sets) are smaller. If we include here those spots that are missing at GD 13 and appear thereafter and then increase in intensity, or alternatively spots that decrease from GD 13 to GD 15 and disappear thereafter, the previously mentioned percentages would be higher (16% for both, increasing and decreasing proteins). In the calculations are only those spots considered that are present on at least two consecutive days of measurement. A considerable portion of spots does not fulfill this criterion; spots present on one gestation day only might be of great importance (and information on them is stored in our database), but we disregard them at present, because there is a chance that some of these spots might be artifactual (improper spot modeling by *Kepler* software) or nonreproducible. For the same reasons, it is not justifiable to interpret those spots that are present on GD 13, absent on GD 15, and present again on GD 17. In future analyses, it might turn out that some of these spots belong to the category of "increasing" or "decreasing" spots; thus, the percentages that we give here are minimal estimates. We anticipate that once having switched (from the carrier ampholytes) to the immobilized pH gradients (Righetti et al., 1988), we will be able to match an even greater number of spots on a single gel. As our estimates are conservative, we believe that the subsets of spots that fulfill the criterion of increasing or decreasing, 16% in both cases, throughout the entire observation period is highly meaningful. Based on the sets of spots with varying intensity detected in our study, we predict that a large number of additional regulatory molecules important in thymic development will be found. We believe that the finding of sets of proteins that are up- or

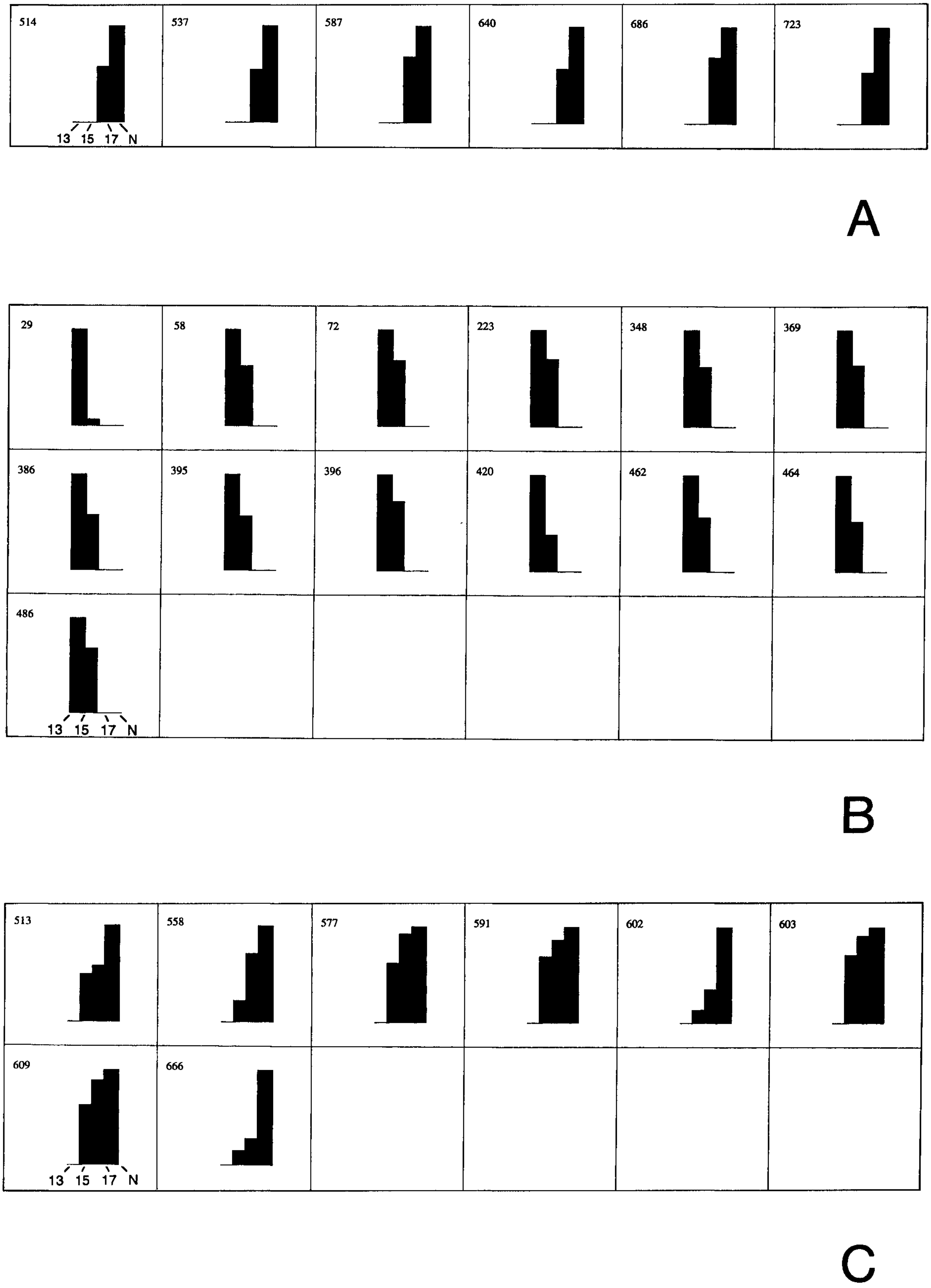

FIGURE 8. Polypeptides in thymuses that are missing at early observation times and appear on GD 15 (part C) or later (part A), and polypeptides that disappear after GD 15 (part B). Figure presentation and histogram layout are identical to that of Fig. 7.

down-regulated during thymus differentiation is of great interest. We will be able to recognize whether similar sets of proteins are altered pertaining to other differentiation processes. Such correlations could unravel new regulatory mechanisms of the immune system. These studies will involve the use of a database under development in our laboratory that will contain the electrophoretic 2D gel patterns of lymphocyte proteins, the so-called proteinpaedia (Lefkovits et al., 1990; Kettman et al., 1994).

We will also proceed with the next stage of the project, namely, to attempt to retrieve and to characterize the coding sequences of the spots identified throughout this work. First, we will make use of our existing cDNA library that was established from BW 5147 cells and we will retrieve at least some of the cDNA clones, those that are shared between thymocytes and the BW 5147 source. Second, we will prepare subtraction libraries of GD 13 and 17 thymocytes, distribute the clones within an ordered library (Lefkovits et al., 1995a) and identify the clones by procedures as described earlier (Béhar et al., 1995; Lefkovits et al., 1995b). Based on the sets of spots with varying intensity detected in our study, we predict that a large number of additional regulatory molecules important in thymic development will be found.

### MATERIALS AND METHODS

### Mice

BALB/c mice received from the Institut für medizinische Forschung (Füllinsdorf, Switzerland) were used in all experiments described. Thymuses were dissected from newborn animals and from fetuses at GD 13, 15, and 17; day 0 was considered the day of appearance of a vaginal plug. The tissue was used for cryosections and to perform biosynthetic labeling.

### Immunohistochemistry

Cryosections were prepared from whole fetuses at GD 13, 15, and 17 and were stained with either anti-Thy-1 (30H12) or anti-MHC class II (P7/7) antibody. The detection was performed by biotinylated goat-anti-rat Ig and streptavidin-peroxidase according to techniques described (Hartmann et al., 1989).

### Sample Preparation and Biosynthetic Labeling

Biosynthetic labeling of thymus lobes *in vitro* was carried out as follows: the whole thymus from 13-day-old fetuses and one lobe from 15- and 17-day-old fetuses were incubated in 200 µl cultures for 15 hr. The medium was RPMI 1640, including 2% of dialyzed FCS, glutamine, 2-ME, antibiotics, and $^{35}$S-methionine obtained from Amersham (Cat. N. SJ204; 1000 Ci/mmol) at 15 µCi/ml. For *in vivo* labeling, day-17 pregnant mice were injected i.v. with 1 mCi of $^{35}$S-methionine and fetuses were excised 5 hr later (Pluschke and Lefkovits, 1990).

### Sample Solubilization

After labeling, the thymic tissue was packed by centrifugal force into Sarstedt tubes (#72.702, Sarstedt,

TABLE 1

Spot Parameters

| | Pattern | | | | |
|---|---|---|---|---|---|
| | GDS 13[a] | GD 15 | GD 17 | Birth | Master[b] |
| Number of spots[c] | 505 | 450 | 550 | 429 | 764 |
| Shared spots, fetal period[d] | 312 | 312 | 312 | | |
| Shared spots, extended period[e] | 272 | 272 | 272 | 272 | |
| Spots volume[f] | | | | | |
| Sum of volumes | 3,337,516 | 3,085,014 | 3,561,898 | 3,323,357 | |
| Mean spot volume | 6,609 | 6,856 | 6,476 | 7,747 | |
| Median spot volume | 4,520 | 3,680 | 3,900 | 4,650 | |
| Interquartile range | | | | | |
| Lower value | 2,050 | 1,850 | 1,870 | 2,490 | |
| Higher value | 8,070 | 7,400 | 7,770 | 8,680 | |
| Volume at 10th percentile | 993 | 956 | 921 | 1,360 | |
| Volume at 90th percentile | 12,700 | 14,200 | 14,900 | 15,900 | |

[a] Thymus tissue collected at indicated gestation day.
[b] Master spot pattern (compare Materials and Methods).
[c] Total number of analyzed spots per pattern.
[d] Spots common to the three fetal patterns (GD 13, 15, and 17, i.e., short observation period).
[e] Spots common to all four patterns (GD 13, 15, 17, and newborn, i.e., extended observation period).
[f] Spot volume (modeled based on the three parameters—*dx*, *dy*, amp—that are the axes of the ellipse and the amplitude—and given in arbitrary units).

Rommelsdorf, Germany) and 60 µl of solubilizing buffer were added with a Hamilton syringe. This buffer consists of 2% Nonidet P-40 (Sigma N-3516), 1% 2ME, and 2% ampholines pH 9–11 (LKB 9–11 1809–146, LKB, Bromma, Sweden) in 9 M urea (Merck), adjusted to pH 9.5 and stored at –70°C.

### Preparation of 2D Gels

The labeled and lysed samples were subjected to isoelectric focusing (IEF) (pH 3–9) followed by 2D-polyacrylamide gel electrophoresis (PAGE) (25 µl of sample were loaded) according to the method originally developed by O'Farrell (O'Farrell, 1975). We used a modification that accommodates 20 samples simultaneously in both IEF and SDS-PAGE in a device designed by Leigh and Norman Anderson, the ISODALT system (Anderson and Anderson, 1978; Lefkovits et al., 1985). The gels were exposed for 12 days to XAR-5 film.

### Scanning and Image Analysis of Autoradiographs

The autoradiographs were scanned with a laser densitometer (constructed at the Federal Technology Institute, Zürich) using 100 µm scanning steps, recording at an OD range 0–1.8. Image analysis was then performed with the *Kepler* software system (Large Scale Biology Corporation, Rockville, MD) installed on a VAXstation 3100.

Gel files are converted into image files and those are processed for noise and streak removal and background correction. The processed image files are converted into spot files by spot modeling and fitting, files in which each spot is defined by five parameters: the $x$ and $y$ coordinates and the spot volume parameters $sx$, $sy$, and amplitude. At this point in the procedure, one spot list pattern is chosen to which all other spot list patterns are compared. Such a list, to which the program assigns a numbering system, is called a master pattern. All spot list patterns are then matched to the master pattern and individual spots receive master spot numbers, congruent to the master spots. Spots on individual patterns that do not occur on the master pattern are transferred into it with a utility provided by the program. At the end of the matching process, the master pattern contains all the spots occurring in each of the patterns. All this information on each and all spots is kept in the *Kepler* database. It keeps track of all images, spot lists, and spot identities and maintains congruence in the whole system.

### Data Analysis

Since each spot on a autoradiograph results upon image analysis in a "modeled" volume, we could use the spot volume as a representation of the abundance of the detected polypeptides. The spot volume data were further analyzed using a spreadsheet program. First, a calibration step had to be carried out keeping the sum of total intensity of all spots in each gel constant, enabling the detection of changes in spot intensities (spot volume data) at the different observation times (GD 13, 15, 17, and birth). A matrix was assembled that contained the spot volume data for all the four time points of the experiment. Two sets of spots were extracted, one to contain the spot volume data of those spots that all occurred on the patterns of the four time points (GD 13, 15, 17, and birth) and another one of those that all occurred on the patterns of just the three fetal time points. These two data sets served to assemble those spots that either increased, decreased, or remained unchanged during the shorter and the extended observation periods. Spots were considered increasing when their intensity was at least 1.125-fold greater at GD 17 or at least 1.25-fold greater at birth compared to their intensity at GD 13. Spots were considered decreasing when their intensity was at least 0.9 fold lower at GD 17 or at least 0.8 fold lower at birth compared to their intensity at GD 13. Spots with volume changes between 0.9- and 1.125-fold between GD 13 and GD 17 or between 0.8- and 1.25-fold between GD 13 and birth were defined as constant. Spots with fluctuating intensities were disregarded, when at the intermittent time point(s) their volumes fell outside a defined range (0.8- and 1.2-fold).

### ACKNOWLEDGMENTS

We gratefully acknowledge the skills of Ms Evelyn Agricola who performed the immunohistochemistry and of Ms Lotte Kuhn who processed the samples, ran the gels, and prepared the autoradiographs. We value the helpful support of Randy Baron and Hubertus Temminck for the work on the VAXstation. We thank Hanspeter Stahlberger for artwork, Ms Beatrice Pfeiffer for photography, and Ms Cynthia Geiger for typing. We are grateful to Drs Beat Imhof and Ed Palmer for critical reading of this manuscript. The Basel Institute for Immunology was founded and is supported by Hoffmann La Roche Ltd., Basel, Switzerland.

*(Received October 5, 1995)*

*(Accepted November 20, 1995)*

### REFERENCES

Anderson N.G., and Anderson N.L. (1978). Analytical techniques for cell fractions. XXI. Two-dimensional analysis of serum and tissue proteins. Multiple isoelectric focusing. Anal. Biochem. **85**: 331–340.

Ardavin C., Wu L., Li C.L., and Shortman K. (1993). Thymic dendritic cells and T cells develop simultaneously in the thymus from a common precursor population. Nature **362**: 761–763.

Béhar G., Coleclough C., Auffray C., and Lefkovits I. (1995). Analysis of individual clones from an ordered cDNA library. Appl. Theor. Electrophor.: In press.

Boyd R.L., and Hugo P. (1991). Towards an integrated view of thymopoiesis. Immunol. Today **12**: 71–79.

Hartmann K.-U., von Eckardstein S., and Darwin E. (1989). Differentiation of epithelial stroma during ontogenesis of the thymus in mice: A speculation on the origin of the thymus as a sensory organ. Thymus **13**: 195–200.

Imhof B.A., Ruiz P., Hesse B., Palacios R., and Dunon D. [1991]. EA-1, a novel adhesion molecule involved in the homing of progenitor T lymphocytes to the thymus. J. Cell. Biol. **114**: 1069–1078.

Ivanov V., Merkenschlager M., and Ceredig R. (1993). Antioxidant treatment of thymic organ cultures decreases NF-kB and TCF1 (alpha) transcription factor activities and inhibits αβ T cell development. J. Immunol. **151**: 4694–4704.

Jotereau F., Heuze F., Salomon V.,' and Gascan H. (1987). Cell kinetics in the fetal mouse thymus: precursor cell input, proliferation, and emigration. J. Immunol. **138**: 1026–1030.

Kettman J., Coleclough C., and Lefkovits I. (1994). Lymphocyte proteinpaedia stage two: T-cell polypeptides from a partitioned cDNA library revealed by the dual decay method. Int. Arch. Allergy Immunol. **103**: 131–142.

Kisielow P., and von Boehmer H. (1995). Development and selection of T cells: facts and puzzles. Adv. Immunol. **58**: 87–209.

Lefkovits I., Frey J.R., and Coleclough C. (1995b). Human lymphocyte cDNA ordered library analyzed by 2D gel electrophoresis 2. Frequency distribution of mRNA populations. Appl. Theor. Electrophor.: 5: 43–47.

Lefkovits I., Frey J.R., Kuhn L., Kettman J.R., Béhar G., Auffray C., Hoffmann J.-P., and Coleclough C. (1995). Human lymphocyte cDNA ordered library analyzed by 2D gel electrophoresis 1. Pooling strategy and matching of gel patterns. Appl. Theor. Electrophor.: 5: 35–42.

Lefkovits I., Kettman J.R., and Coleclough C. (1990). A strategy for founding a global lymphocyte proteinpaedia and gene catalogue. Immunol. Today **11**: 157–162.

Lefkovits I., Kuhn L., Valiron O., Merle A., and Kettman J. (1988). Toward an objective classification of cells in the immune system. Proc. Natl Acad. Sci. USA **85**: 3565–3369.

Lefkovits I., Young P., Kuhn L., Kettman J., Gemmell A., Tollaksen S., Anderson L., and Anderson N. (1985). Use of large-scale two-dimensional ISODALT gel electrophoresis system in immunology. *In:* Immunological Methods, vol. 3, Lefkovits I, and Pernis B., Eds. (Orlando, FL: Academic Press), pp. 163–185.

O'Farrell P.H. (1975). High resolution two-dimensional electrophoresis of proteins. J. Biol. Chem. **250**: 4007–4021.

Palacios R., and Samaridis J. (1991). Thymus colonization in the developing mouse embryo. Eur. J. Immunol. **21**: 109–113.

Pluschke G., and Lefkovits I. (1990). Analysis of two-dimensional gel electrophoretic protein patterns upon in vivo labeling of mice. *In:* Immunological Methods, vol. 4, Lefkovits I, and Pernis B., Eds. (Orlando, FL: Academic Press), pp. 153–164.

Righetti P.G., Chiari M., and Gelfi C. (1988). Immobilized pH gradients: Effect of salts, added carrier ampholytes and voltage gradients on protein patterns. Electrophoresis **9**: 65–73.

Scollay R. (1980). Thymus cell migration: Quantitative aspects of cellular traffic from the thymus to the periphery in mice. Eur. J. Immunol. **10**: 210–218.

Scollay R., and Godfrey D.I. (1995). Thymic emigration: conveyor belts or lucky dips? Immunol. Today **16**: 268–273.

Sen J., Shinkai Y., Alt F.W., Sen R., and Burakoff S.J. (1994). Nuclear factors that mediate intrathymic signals are developmentally regulated. J. Exp. Med. **180**: 2321–2327.

Tough D.F., and Sprent J. (1995). Thymic emigration—A reply. Immunol Today **16**: 273–274.

Van Ewijk W., Shores E.W., and Singer A. (1994). Crosstalk in the mouse thymus. Immunol. Today **15**: 214–217.

Xie X., and Palacios R. (1994). Cloning and expression of a new mammalian chaperonin gene from a multipotent hematopoietic progenitor clone. Blood **84**: 2171–2174.

